# Two-center clinical validation and quantitative assessment of respiratory triggered retrospectively cardiac gated balanced-SSFP cine cardiovascular magnetic resonance imaging in adults

**DOI:** 10.1186/s12968-018-0467-6

**Published:** 2018-06-28

**Authors:** Amol S Pednekar, Hui Wang, Scott Flamm, Benjamin Y. Cheong, Raja Muthupillai

**Affiliations:** 1 Department of Radiology, Texas Children’s Hospital, 6701 Fannin Street, Suite D470.09, Houston, TX 77030-2399 USA; 2 Philips Healthcare, Gainesville, FL USA; 3 Department of Diagnostic Radiology, Cleveland Clinic, Cleveland, OH USA; 4 Department of Radiology, Baylor St. Luke’s Medical Center, Houston, TX USA

**Keywords:** Cardiovascular magnetic resonance, Left ventricular function, Free breathing respiratory-triggered cine

## Abstract

**Background:**

Breath-hold (BH) requirement remains the limiting factor on the spatio-temporal resolution and coverage of the cine balanced steady-state free precession (bSSFP) cardiovascular magnetic resonance (CMR) imaging. In this prospective two-center clinical trial, we validated the performance of a respiratory triggered (RT) bSSFP cine sequence for evaluation of biventricular function.

**Methods:**

Our study included 23 asymptomatic healthy subjects and 60 consecutive patients from Institute A (*n* = 39) and Institute B (*n* = 21) referred for a clinically indicated CMR study. We implemented a RT sequence with a respiratory synchronized drive to steady state (SS) of bSSFP signal, before the commencement of image data acquisition with prospective cardiac arrhythmia rejection and retrospectively cardiac gated reconstruction in real-time. Left (LV) and right (RV) ventricular function and LV mass were evaluated by using RT-bSSFP and conventional BH-bSSFP sequences with one cardiac cycle for SS preparation keeping all the imaging parameters identical. The performance of the sequences was evaluated by using quantitative and semi-quantitative metrics.

**Results:**

Global LV and RV functional parameters and LV mass obtained from the RT-bSSFP and BH-bSSFP sequences were in good agreement. Quantitative metrics designed to capture fluctuation in SS signal intensity showed no significant difference between sequences. In addition, blood-to-myocardial contrast was nearly identical between sequences. The combined clinical score for image quality was excellent or good for 100% of cases with the BH-bSSFP and 83% of cases with the RT-bSSFP sequence. The de facto image acquisition time for RT-bSSFP was statistically significantly longer than that for conventional BH-bSSFP (7.9 ± 3.4 min vs. 5.1 ± 2.6 min).

**Conclusions:**

Cine RT-bSSFP is an alternative for evaluating global biventricular function with contrast and spatio-temporal resolutions that are similar to those attained by using the BH-bSSFP sequence, albeit with a modest time penalty and a small reduction in image quality.

## Background

In patients with cardiovascular disease, ventricular volume, mass, and ejection fraction (EF) are powerful predictors of prognosis [1–5]. In several studies of individuals with normal and abnormal ventricles, cardiovascular magnetic resonance (CMR) has been used to evaluate biventricular volume, mass, and EF with high accuracy and low inter- and intra-observer variability [6–9]. For the accurate measurement of ventricular functional indices in CMR, it is necessary to have: a) sufficient and consistent blood-to-myocardium contrast throughout the cardiac cycle to reliably define endocardial contour, b) adequate spatio-temporal resolution to accurately capture end-diastole and end-systole, and c) immunity to cardiorespiratory motion and pulsatile blood flow. In routine clinical CMR practice, cine balanced steady-state free precession (bSSFP) is a sequence of choice for evaluation of ventricular function [10] as it offers the highest signal-to-noise ratio (SNR) per unit time of all other CMR imaging sequences along with a T_2_/T_1_-weighted image contrast [11]. In order to obtain diagnostic image quality, the bSSFP sequence mandates the shortest possible repetition time (TR) to minimize banding artifacts [11], high flip angle for adequate blood to myocardial contrast [12], and uninterrupted radiofrequency (RF) excitations to maintain the magnetization at steady-state (SS) to elude contrast variability [13] and artifacts [14, 15]. In addition, cine bSSFP acquisition employs retrospective cardiac gating to obtain the entire cardiac cycle along with prospective arrhythmia rejection. In clinical practice breath-holds (BH) are used to mitigate respiratory motion artifacts. Respiratory suspension for sufficient duration is not feasible in sedated patients and patients with impaired breath-holding capacity. Therefore, it would be of clinical interest to obtain cine bSSFP images without the constraint of breath holding while retaining the necessary features described above.

Approaches to combat artifacts introduced by respiratory motion in cine CMR imaging fall in three broad categories: (1) rapid imaging with data undersampling [16–18]; (2) respiratory motion synchronized data acquisition using signals from an external respiratory bellows or RF navigator positioned over the lung-diaphragm interface [19–23]; and (3) self-gated motion compensation methods [24–26]. Combinations of data undersampling and self-gating are being explored for respiratory motion-state cine imaging [27]. Some of these approaches require contrast administration [18, 24, 28], or prospective cardiac gating [16, 17] and undersampling methods may require considerably longer reconstruction times and/or advanced hardware capabilities [16, 18, 24, 25, 27]. The dual RF navigator gated approach was limited to partial acquisition of the cardiac cycle [19]. In contrast, respiratory synchronized acquisition methods using bellows or diaphragmatic RF navigators permit retrospective cardiac gating and real-time reconstruction of bSSFP cine sequences, which are used in clinical practice without imposing any hardware or reconstruction burden [20–23]. Furthermore, these respiratory motion synchronized techniques allow the flexibility to be seamlessly combined with undersampling strategies such as compressed sensing that may become clinically available in the future.

We have previously demonstrated respiratory synchronized bSSFP cine acquisitions using both respiratory bellows and diaphragmatic RF navigator, in a healthy cohort study [20]. These implementations require SS to be reached before the acquisition of cardiac gated cine data to ensure uniform signal and contrast throughout the cardiac cycle. The bellows based respiratory triggered (RT) implementation was validated in a pediatric population [22]. Similar approaches using respiratory bellows and diaphragmatic RF navigator for respiratory synchronized bSSFP cine acquisition have also been reported [21, 23]. However, these implementations use the available time between the respiratory trigger and the subsequent R-top for SS preparation. This can cause artifacts because the variable time interval may not be long enough to attain SS in certain respiratory cycles [21]. In this manuscript we describe a two-center clinical validation of a bellows based RT-bSSFP in an adult population with rigorous quantitative assessment of the temporal stability of the myocardial signal as well as blood-to-myocardial contrast.

## Methods

### Numerical simulation

Numerical simulations were performed in MATLAB (The MathWorks™ Inc., Natick, Massachusetts, USA), to determine the minimum number of RF excitations required for myocardial tissue to reach SS in a bSSFP sequence starting with an α/2 preparation pulse, followed after a time period of TR/2 by successively alternating ±α excitation pulses every TR. Temporal evolution of myocardial magnetization from the unperturbed state to SS with uninterrupted RF excitations, characteristic of the bSSFP sequence, was numerically simulated with the following parameters: magnetic field strength, 1.5 T ± 3 ppm; TR/TE/flip angle, 3 ms/1.5 ms/65°; T_1_, 950 ms; T_2_, 50 ms. The steady state magnetization (M_SS_) was defined as magnetization where the rate of change of magnetization was close to zero (< 0.5%).

### Cine bSSFP CMR with respiratory triggering (RT)

**Fig. 1:**
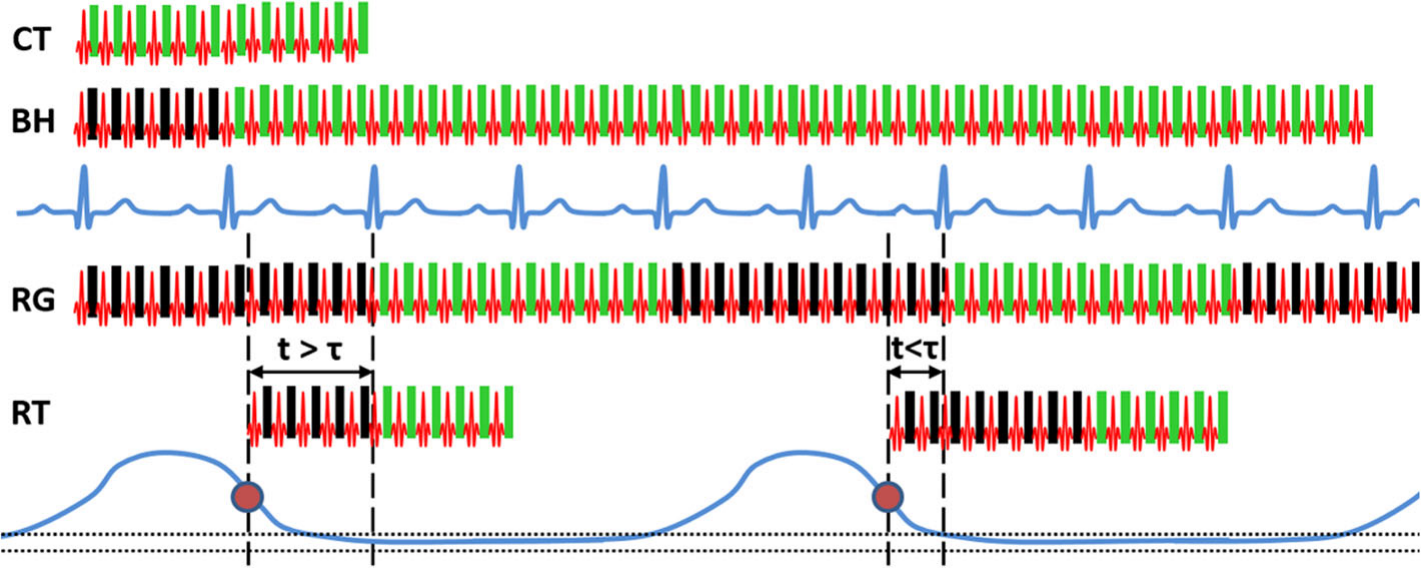
Steady-state (SS) magnetization preparation methods for cardiac-gated balanced steady-state free precession (bSSFP) cine. **CT**: In simple cardiac-triggered (CT) acquisition with no SS preparation, all data acquired after the detection of a valid cardiac trigger are accepted for image formation (green boxes). **BH**: In conventional breath-hold (BH) SS preparation, data acquired during the first RR interval are discarded (black boxes). All subsequent data acquired with cardiac gating during suspended respiration are used for image formation. **RG**: In the respiratory-gated (RG) method, uninterrupted radiofrequency (RF) excitations applied throughout the acquisition act as the means for SS retention, and only those cardiac-gated data that are acquired when the respiratory bellows signal falls within the user-defined threshold (horizontal dotted lines) are accepted for image formation. **RT**: In the respiratory-triggered (RT) approach proposed in this paper, RF excitation commences after the detection of a respiratory trigger (e.g., inspiration or expiration), and only data acquired during an RR interval that lags the respiratory trigger point by at least a predefined duration of τ is used for image formation. Unlike RG, RF excitation ceases after the RR interval in which image data are acquired. Electrical signals from the cardiac leads and respiratory bellows are shown in blue. Note that this algorithm is fully compatible and is implemented with prospective rejection of cardiac arrhythmias and retrospective cardiac gating. Phase-encoding steps for the multi-phase segmented k-space acquisition are changed only after successful acceptance of the data. Red dot, expiration trigger; RF, radio frequency; black rectangle, data are discarded; green rectangle, data are accepted for further processing; t, time after respiratory trigger; τ, time to attain steady state; dotted line, respiratory acceptance window

A commercially available BH cine bSSFP sequence with retrospective cardiac gating and prospective arrhythmia rejection was modified to implement a free-breathing RT sequence on clinical scanner (Achieva, Philips Healthcare, Best, the Netherlands). A schematic showing the building blocks of this technique, described previously [22], is shown in Fig. 1. In the RT sequence, the RF excitations started immediately after the trigger signal (could be set to an arbitrary delay from inspiration or expiration trigger point) derived from the respiratory bellows unmodified from the commercial implementation on the scanner. The data acquisition corresponding to the initial RF excitations were discarded (dummy excitations), for a minimum user-prescribed time (τ), to drive to M_SS_. The subsequent arrival of the first cardiac R-top (at time point after the trigger > τ) was used as a cardiac synchronization point to accept data acquisition of the segment of phase-encoding steps in *k*-space in a multi-phase manner during that cardiac cycle. Upon arrival of the subsequent R-top, RF excitations were terminated, and the commercially available real-time arrhythmia rejection algorithm either accepted or rejected the data. In the case of rejection, the same phase-encoding segment was re-acquired. This process was repeated until the entire *k*-space was acquired, after which the retrospective cardiac gating reconstruction algorithm performed the required nonlinear stretching of variable R-R intervals to reconstruct cine images.

### Two-center clinical validation

The prospective clinical validation studies were performed at Institute A and Institute B in patients referred for a clinical CMR for various indications. Institute A implemented the RT sequence and Institute B, located 2000 km away from Institute A, used this investigational sequence without supervision from Institute A. Institute A recruited 23 asymptomatic subjects (10 men). The mean age of these healthy subjects was 44 years (range, 24–60 years). At Institute A, 39 consecutive patients (21 men) were enrolled in the study. The mean age of these patients was 50 years (range, 19–82 years). At Institute B, 21 consecutive patients (12 men) were enrolled in the study. The mean age of these patients was 47 years (range, 23–86 years). All patients underwent biventricular function evaluation as a part of routine clinical protocol to evaluate the following conditions: left and right ventricular (LV, RV) cardiomyopathies (31), myocardial viability (12), valvular function (9), pericarditis (7), and congenital heart disease (1). This study was approved by the Institutional Ethics Committee of Catholic Health Initiative’s Institute for Research and Innovation under evolution MRI techniques protocol and complied with the Health Insurance Portability and Accountability Act of 1996. All subjects gave written informed consent before being enrolled in the study. All healthy subject and patient imaging was performed at 1.5 T CMR scanner (Achieva, Philips Healthcare, Best, the Netherlands) with a 5-element or 32-element phased-array surface coil for signal reception and vector-cardiographic (VCG) gating, with respiratory bellows placed at mediastinum. Authors employed by the CMR scanner manufacturer were not part of the clinical patient data acquisition and image quality assessment.

### Cardiovascular magnetic resonance imaging protocol

In all 83 study participants, scout images of the thoracic cavity were obtained along the three orthogonal planes by using a non-VCG-gated bSSFP technique. With these single-phase scout scans, a series of VCG-gated cine images were acquired during suspended respiration (BH technique) in the following order: a two-chamber view, a four-chamber view, and a series of 12 to 14 contiguous short-axis slices covering the entire LV from the apex to the base (the level of the mitral valve annulus). Identical imaging parameters and short-axis slice prescription were used with the RT acquisition. Patients were completely blinded to the imaging sequences and were not given any special instructions for breathing during RT technique.

The imaging parameters for both BH and RT cine bSSFP techniques were as follows: TR/TE/flip angle = 2.5–2.7 ms/1.25–1.35 ms/65°; acquired voxel size = 1.7–2.0 × 1.6–2.0 × 8 mm^3^; SENSitivity Encoding (SENSE) acceleration factor = 1.3–1.9; temporal resolution = 40-50 ms. In the case of BH, each cine slice was acquired during suspended respiration (6–8 R-R intervals/slice, 10–16 heartbeats brath hold time for 2 slices). In the case of RT, a user controllable parameter, minimum time (τ) to drive SSFP signal to M_SS,_ was kept fixed at the default value of 450 ms for all the subjects. For the purpose of evaluating the impact of signal evolution during approach to SS on image quality, additional single-shot cardiac-triggered cine bSSFP (Fig. 1) images without preparation for SS were also acquired in 10 volunteers in a mid-ventricular slice with other imaging parameters identical to those used for BH and RT sequences.

### Image analysis

All data were anonymized, randomized, and transferred to a commercial post processing workstation (ExtendedWorkSpace, Philips Healthcare).

#### Quantitative assessment of signal variation

For 23 healthy subjects, regions of interest (ROI) were drawn in the liver, myocardium, and blood pool at mid-ventricular level and were propagated across all cardiac phases in all cine bSSFP sequences. A histogram-based analysis was used to define two quantitative metrics designed to capture the extent of signal intensity (SI) variation across the cardiac cycle, as well as the percent duration of the cardiac cycle (PCC) during which tissue magnetization was close to the theoretically predicted M_SS_. The signal intensity of ROIs was normalized to the minimum SI across the cardiac cycle. To construct a histogram, normalized signal intensity values were plotted against the PCC spent at that normalized intensity level. Cumulative density function of normalized signal intensity of myocardial ROI over the cardiac cycle was computed for all healthy subjects. The ratio of the normalized signal intensity values at 95 and 5% of cumulative density function was computed as a metric to characterize the extent of signal intensity variation during the cardiac cycle. A normalized signal intensity of 1.25, which corresponded to M_SS_ based on the simulations, was used to compute the PCC spent in SS. The blood-myocardial contrast (BMC) was normalized to myocardial signal intensity. Box plots were used to evaluate the signal intenisty variation (SIV), the PCC spent in SS by the liver and myocardium, and BMC; in these plots, non-overlapping notches indicate that the medians of the two groups differed at the 5% significance level. In addition, paired two-sided Student’s *t*-statistics for SIV, PCC, and BMC were used to compare among cardiac triggered, BH, and RT sequences.

#### Quantitative assessment of ventricular measurements

Two independent CMR readers, each with at least a decade of experience in clinical CMR, independently drew the ventricular contours necessary to compute LV and RV volumetric indices (end-diastolic volume, end-systolic volume, and EF) and LV mass. Descriptive statistics of each of these parameters for 60 patients using BH and RT are reported as mean ± standard deviation in absolute values and percentages. Bland-Altman analysis [29] and paired two-sided Student’s *t*-statistics were used to compare each of these parameters computed using the BH sequence with those computed using the RT sequence in 60 patients. Inter-observer variability for each of these parameters was assessed using Bland-Altman analysis in both BH and RT acquisitions in 60 patients.

#### Image quality assessment

For 60 patients, an independent expert with 12 years of experience in CMR who was blinded to the study design reviewed the stack of short-axis images. The image quality between the two techniques was compared by using the clinical scores, which were based on three parameters: BMC, endocardial-edge definition, and motion artifact. Each parameter was graded on a scale from 1 (non-diagnostic) to 5 (excellent) on review of all the images in the short-axis stack. In cases where all but one or two of the slices were of high quality, the clinical score was lowered for the entire stack. Details of the scoring criteria are provided in Table 1. For each of the three scoring criteria considered in the study, the percentage of clinical subjects that received a range of clinical scores was plotted as a bar graph. In addition, a similar bar graph was constructed with the combined score, computed as the equal weight average of the three scores, to underscore the overall performance of the technique on the basis of all three criteria. One-sided Wilcoxon signed rank tests were performed on clinical scores assigned to the BH and RT techniques.

**Table 1:** Clinical score criteria

Score	Blood-to-myocardial contrast	Endocardial edge definition	Motion artifact
5 - Excellent	Blood pool is hyper intense with excellent contrast against the myocardium; myocardium is uniformly bright throughout the cardiac cycle with little evidence of flashing.	Papillary and endocardial trabeculae are clearly visible in the bright backdrop of the blood pool.	Image is nearly artifact free.
4 - Good	Blood pool is significantly brighter than the myocardium, or myocardial signal intensity is fairly uniform throughout the cardiac cycle.	Papillary and endocardial trabeculae are visible but somewhat blurred during the cardiac cycle.	Some motion artifact is present but does not affect overall image quality.
3 - Moderate	Image is of diagnostic quality but features significant loss of blood to myocardial contrast or noticeable variation in myocardial signal throughout the cardiac cycle.	Myocardial walls are barely distinguishable from endocardial trabeculae.	Motion artifacts are visible, but image is still of diagnostic quality.
2 - Poor	Blood-to-myocardial contrast is poor, but the image is still of diagnostic quality.	Myocardial walls and endocardial trabeculae are significantly blurred.	Images are nearly nondiagnostic with significant artifacts.
1 - Nondiagnostic	Blood-to-myocardial contrast is poor; image was deemed nondiagnostic.	Blood-to-myocardial edge definition is poor; image was deemed nondiagnostic.	Image is of nondiagnostic quality.

## Results

### Numerical simulation

**Fig. 2:**
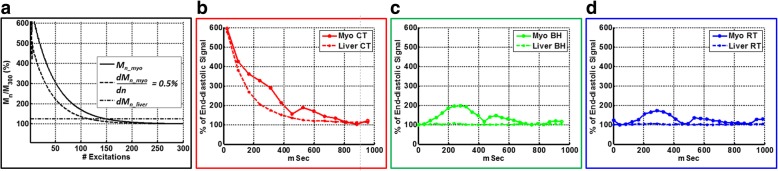
Signal evolution in balanced steady-state free precession (bSSFP) cine sequences from simulation and healthy subject 3. **a**: Numerical simulation of the signal evolution of myocardium (T_1_ = 950 ms, T_2_ = 50 ms) and liver (T_1_ = 600 ms, T_2_ = 45 ms) with continuous radiofrequency (RF) excitation (flip angle = 65^o^; TE = 1.5 ms; TR = 3 ms) in bSSFP sequence. Magnetization steady state (M_SS_) is defined as the temporal marker for when the rate of change of magnetization between successive excitations is less than 0.5% (myocardium, *n* = 140; liver, *n* = 115, 1.25*M_300_). As shown, the initial magnetization level after the first excitation is 6 (liver) to 8 (myocardium) times higher than at *n* = 300. **b**: For cardiac-triggered (CT) sequence (red lines) in Volunteer 3, experimentally measured intensity normalized to the minimum signal intensity value (NI) across cardiac phases (reflecting the M_SS_ of a given tissue) is shown for given regions of interest. The cardiac triggered sequence of the liver and myocardial tissues spans a large variation before reaching steady state, as predicted by theory. **c**: For conventional breath-hold (BH) sequence (green lines) in healthy subject 3, non-moving liver SI shows little variation from M_SS_, but a slight modulation of myocardial signal intensity is seen, especially during the systolic period because of through-plane motion when fresh spins move in and out of the slice of excitation. **d**: For respiratory-triggered (RT) sequence (blue lines) in healthy subject 3, liver and myocardial NI mimic the same pattern as conventional BH acquisitions

**Fig. 3:**
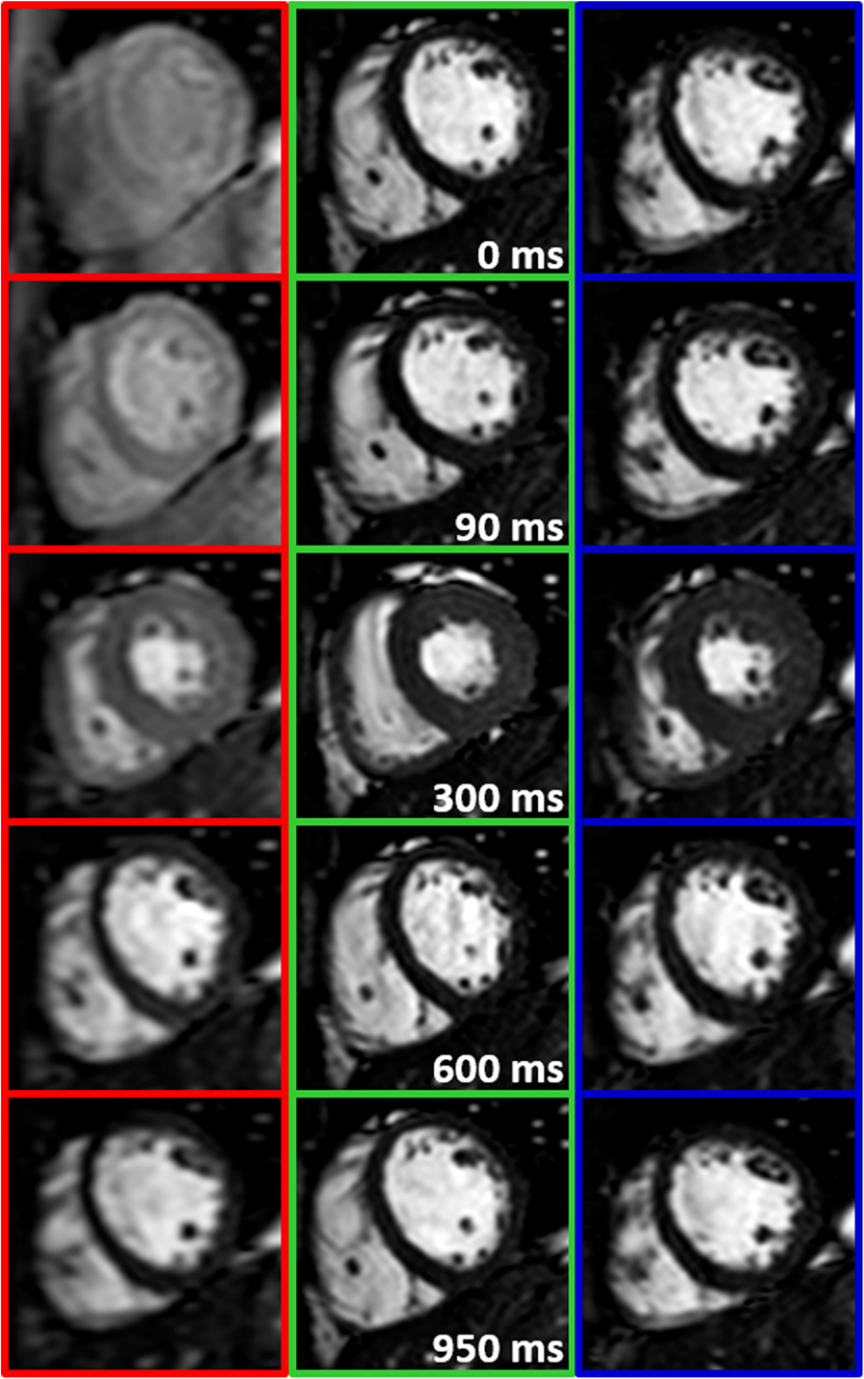
Representative cardiac-gated balanced steady-state free precession (bSSFP) images from healthy subject 3 with no steady-state (SS) preparation for cardiac-triggered (CT) sequence (red), 1-RR SS preparation for breath-hold (BH) sequence (green), and SS preparation for respiratory-triggered (RT) sequence (blue). Unlike in the case of CT sequence, in the RT sequence, tissue magnetization attains steady-state in a manner analogous to the conventional BH sequence with 1 R-R preparation

Numerical simulations showed that the signal intensity for the myocardium could be 6 to 8 times that of the M_SS_ after the first few RF excitations, and as many as 140 continuous RF excitations (with a flip angle of 65^o^ at a repetition time of 3 ms) were required to reach myocardial M_SS_. After the rate of change of myocardial magnetization fell below 0.5%, the magnetization corresponded to 125% of the M_SS_ predicted by the analytical expression for the steady state signal (Fig. 2a). For healthy subject 3, liver and myocardial signal intensity curves with cardiac triggering (no dummy excitations) confirmed the requirement of continuous RF excitation for 350 ms and 450 ms (at TR interval of 3 ms), respectively, for the liver and myocardium (Fig. 2b) to attain steady signal intensity levels. Liver and myocardial signal intensity curves obtained with the RT sequence (dummy excitations for a duration of 450 ms) matched closely with those obtained using the BH sequence (dummy excitations for a duration of one R-R interval), confirming the attainment of M_SS_ after 140 continuous RF pulses, as indicated by the numerical simulation (Fig. 2). Representative clinical images from healthy subject 3 are shown for all three techniques in Fig. 3.

### Quantitative assessment of signal variation

**Fig. 4:**
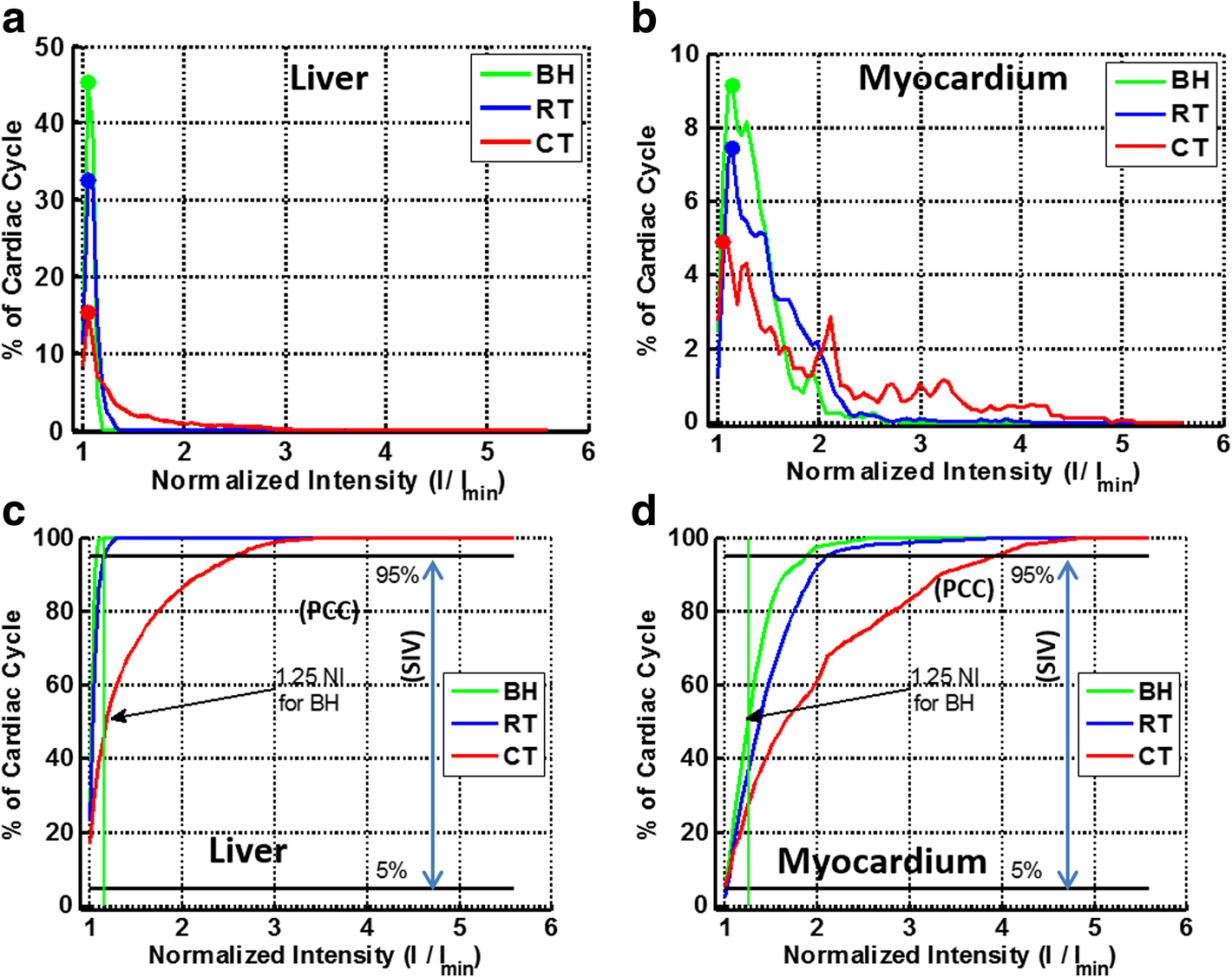
Extraction of quantitative metrics from histogram analysis of liver and myocardial normalized intensity (NI, mean of all subjects) across the cardiac cycle for three steady state (SS) preparations. To generate a histogram, the normalized signal intensity of liver **a** and myocardial **b** signal intensity (SI) is plotted against the fraction of the cardiac cycle occupied by that normalized signal intensity level. When data are plotted in this manner, the mode of the histogram (circles) indicates the in vivo steady state normalized signal intensity level for each of the SS preparation methods. **c**, **d**: Cumulative density functions of histograms plotted in A and B are used to calculate the percentage of cardiac cycle spent below a specific normalized signal intensity level. Total percentage of cardiac cycle (PCC) spent close to steady-state level is defined as cumulative density function at 1.25*normalized signal intensity (vertical line) level (refer to legend in Fig. 2). Temporal signal intensity variation (SIV) is defined as the ratio of normalized signal intensity values at 95 and 5% (horizontal lines) of cumulative density function

**Fig. 5:**
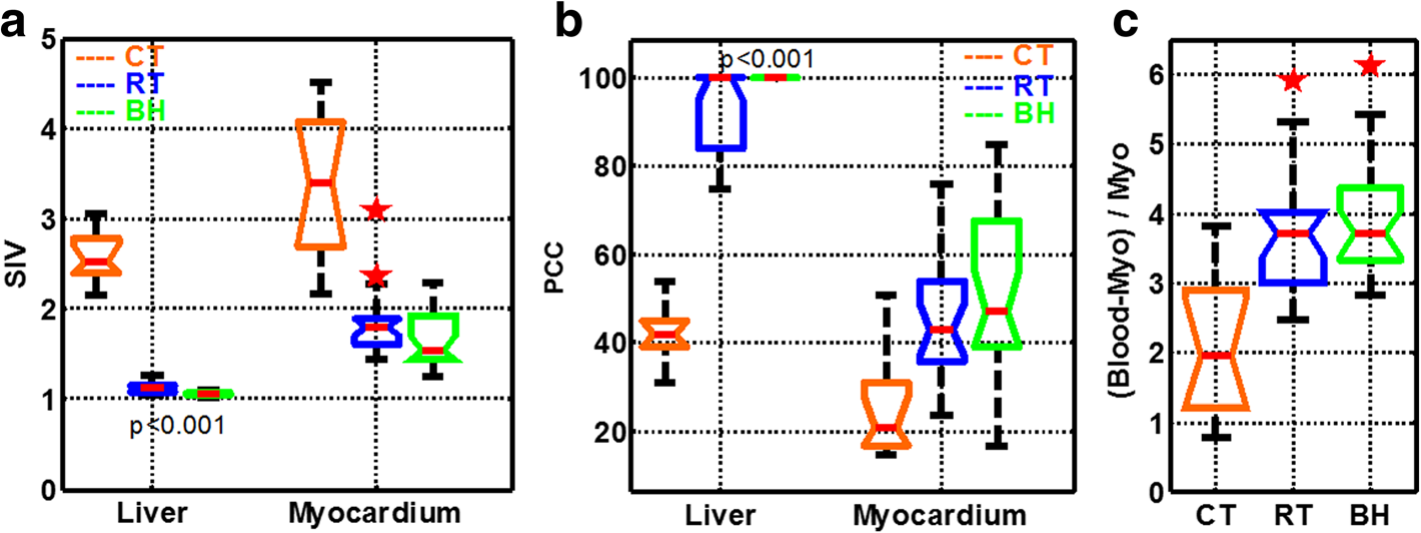
Box-plot analyses of normalized intensity (NI) variation over a cardiac cycle. **a**: Signal intensity variation (SIV) of myocardium was similar between breath-hold (BH) and respiratory-triggered (RT) techniques in study participants (*p* = nonsignificant [NS]), but SIV of myocardium and liver was significantly higher for the CT technique than for either the BH or RT technique (*p* < 0.001); **b**: Percentage of cardiac cycle (PCC) of myocardium was similar between BH and RT techniques (*p* = NS), but the PCC of myocardium and liver was significantly lower for the cardiac triggered technique than for either the BH or RT technique (*p* < 0.001); **c**: The blood-to-myocardial contrast (BMC) of the BH and RT techniques were similar (*p* = NS), but the BMC of the cardiac triggered technique was lower than that of either the BH or RT technique (*p* < 0.001). Although the liver and myocardium have similar tissue relaxation parameters, SIV and PCC were significantly different (*p* < 0.001) between the two tissues for both BH and RT because of through-plane motion of the myocardium. Non-overlapping notches indicate that the medians of the two groups differ at the 5% significance level. Outliers beyond 1.5 times the interquartile distance are indicated with five-point stars in red. *P*-values derived from paired t-test are labeled next to box plots where non-overlapping notches are unclear

Using histogram analysis, we showed the mean normalized signal intensity among all 23 healthy subjects for liver and myocardial ROIs for the BH and RT sequences, and among 10 healthy subjects for cardiac triggered sequences (Fig. 4a-b). When compared to the histogram mode of normalized signal intensity of liver tissue, the histogram mode of normalized signal intensity of the myocardium occupied a much smaller fraction of the cardiac cycle for all three techniques. The cumulative density function of myocardial normalized signal intensity indicated that, on average, the BH and RT techniques spent more than 95% of the cardiac cycle below a normalized signal intensity of 2, whereas for cardiac triggering, that value was 4 (Fig. 4d). Notably, the quantitative metrics of SIV, BMC, and PCC spent in SS for the cardiac triggered technique were significantly different from those for the BH and RT techniques (*p* < 0.001) (Fig. 5). In contrast, the difference in SIV and PCC spent in SS by myocardium was not statistically significant between the BH and RT techniques (Fig. 5a, b). Similarly, BMC was also not statistically different between the BH and RT techniques (Fig. 5c). The differences in SIV and the PCC between the liver and myocardium were statistically significant (*p* < 0.001) for both the BH and RT techniques because of through-plane motion of the myocardium (Fig. 5a, b).

### Quantitative assessment of ventricular measurements

**Fig. 6:**
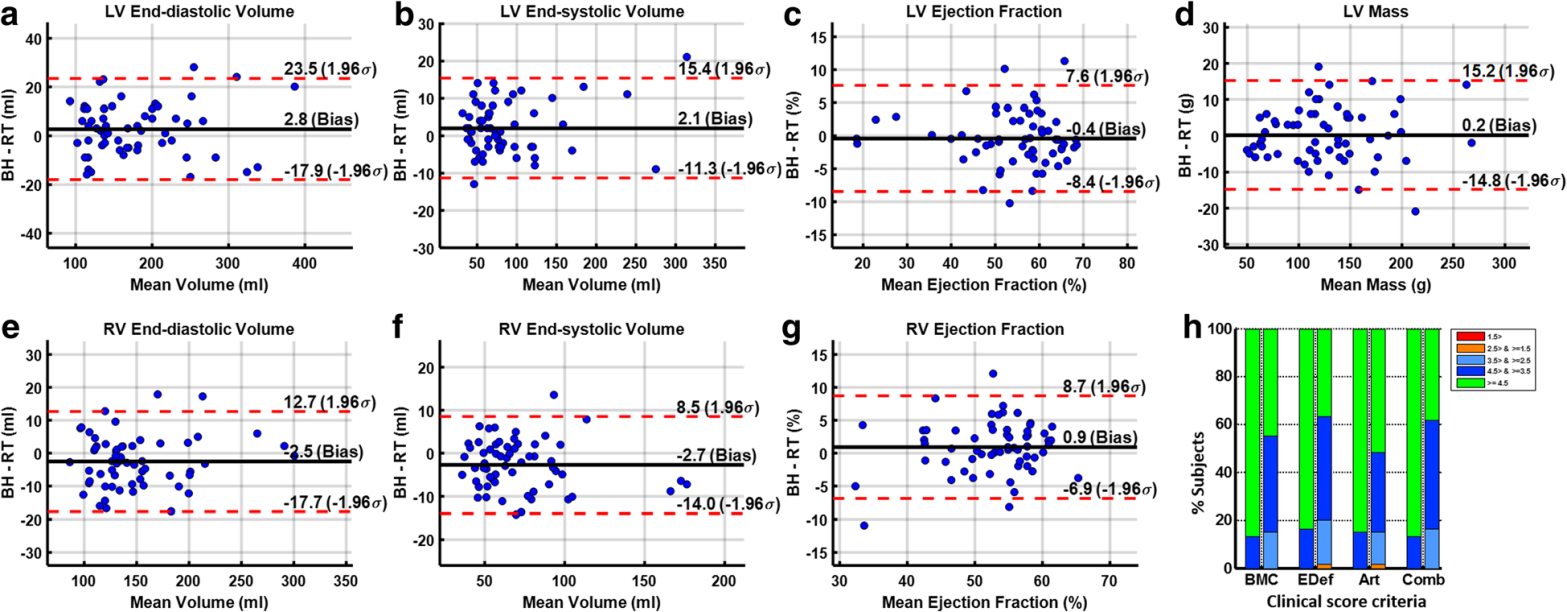
Bland-Altman plots (**a**-**g**) comparing left and right ventricular (LV, RV) volumetric indices and LV mass between BH and RT, and bar-plot analysis (**h**) for combined clinical scores for criteria defined Table 1 (For each criteria, scores for BH are plotted on the left, and RT are plotted on the right). H: Percentage of patients that had clinical scores of excellent, good, moderate, or poor for criteria related to blood-to-myocardial contrast (BMC), endocardial edge definition (Edef), and artifacts (Art). The combined clinical score is the equal-weights average of the three scores, which underscores the overall performance of the technique. **BH**: breath-hold (BH), with a dummy excitation duration of one R-R interval; **RT**: prospectively respiratory-triggered (RT) with dummy excitation duration of 450 ms

The RT sequence ran successfully in all 60 patients. The mean heart rate was 74 bpm (range 43–116), and the mean respiratory rate was 18 bpm (range 12–20). Total image acquisition time for RT was significantly longer than that for conventional BH (7.9 ± 3.4 min vs. 5.1 ± 2.6 min) (*p* < 0.001). In two cases, RT scan time was three times longer than that of the BH sequence because of specific breathing patterns. Table 2 provides descriptive statistics of the LV and RV volumetric indices and LV mass computed using BH and RT sequences in the 60 patients. Figure 6 depicts Bland-Altman plots comparing each of these parameters between BH and RT in 60 patients. Inter-observer variability for each of these parameters in both BH and RT acquisitions in 60 patients is reported in Table 3. For all the parameters the bias and limits of agreement between BH and RT sequences are in good agreement with inter-observer variability in both BH and RT sequences.

**Table 2:** Left and right ventricular volumetric indices and LV mass and difference in their values between breath-hold sequence and respiratory-triggered sequence in patients

	LV EDV (ml)	LV ESV (ml)	LV EF (%)	LV Mass (g)	RV EDV (ml)	RV ESV (ml)	RV EF (%)
BH	177.5 ± 65.8	86.7 ± 56.7	53.9 ± 11.5	126.6 ± 49.9	147.3 ± 45.9	70.2 ± 29.5	53.1 ± 7.6
RT	174.8 ± 65.1	84.6 ± 55.3	54.3 ± 11.8	126.4 ± 49.6	149.8 ± 45.0	73.0 ± 30.3	52.2 ± 7.0
BH-RT	2.8 ± 10.6	2.1 ± 6.8	−0.4 ± 4.1	0.2 ± 7.7	−2.5 ± 7.8	− 2.7 ± 5.7	0.9 ± 4.0
BH-RT / BH (%)	1.4 ± 6.5	2.2 ± 9.5	− 0.9 ± 7.7	−0.2 ± 6.2	−2.1 ± 5.2	−4.5 ± 7.2	1.72 ± 8.9
BH-RT (p)	0.047	0.022	0.450	0.822	0.105	< 0.001	0.072

All values from observer 1

*BH* breath-hold, *EDV* end-diastolic volume, *EF* ejection fraction, *ESV* end-systolic volume, *LV* left ventricle, *p p* value from paired t-test, *RT* respiratory triggered, *RV* right ventricle

**Table 3:** Inter-observer agreement for left and right ventricular volumetric indices and LV mass obtained with breath-hold sequence and respiratory-triggered sequence in patients

	LV EDV (ml)	LV ESV (ml)	LV EF (%)	LV Mass (g)	RV EDV (ml)	RV ESV (ml)	RV EF (%)
BH (O_1_-O_2_)	0.3 ± 10.4	1.3 ± 9.8	−0.7 ± 6.2	1.6 ± 8.3	4.6 ± 6.7	0.4 ± 5.8	1.4 ± 2.8
RT (O_1_-O_2_)	−0.1 ± 11.4	−2.0 ± 8.5	1.9 ± 5.0	− 0.2 ± 8.9	2.7 ± 7.7	1.0 ± 5.6	0.4 ± 3.1
BH (O_1_-O_2_)/ O_1_ (%)	−0.8 ± 8.1	−0.7 ± 16.0	−2.4 ± 12.3	− 0.1 ± 8.4	3.1 ± 4.5	0.2 ± 7.2	2.4 ± 5.5
RT (O_1_-O_2_)/ O_1_ (%)	−0.2 ± 7.3	−6.1 ± 16.3	2.7 ± 8.4	−1.9 ± 9.7	1.6 ± 4.9	0.7 ± 6.5	0.5 ± 6.6

*BH* breath-hold, *EDV* end-diastolic volume, *EF* ejection fraction, *ESV* end-systolic volume, *LV* left ventricle, *O*_1_ observer 1, *O*_2_ observer 2, *RT* respiratory triggered, *RV* right ventricle

### Image quality assessment

**Fig. 7:**
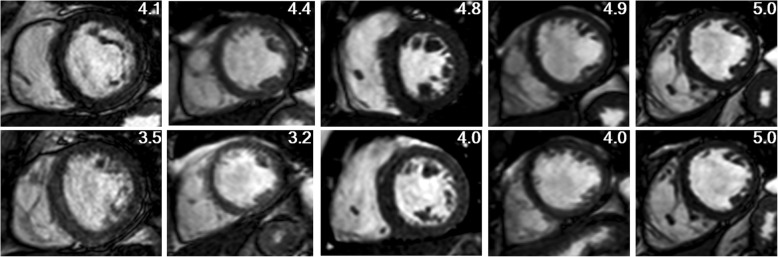
A panel of representative breath-hold (BH) (top) and respiratory-triggered (RT) (bottom) cardiac balanced steady-state free precession (bSSFP) images from patients with a combined clinical score for BH images ranging from 4.1 (good) to 5 (excellent) and a combined clinical score for corresponding RT images ranging from 3.2 (moderate) to 5 (excellent)

The combined clinical score was excellent (87%) to good (13%) for BH and excellent (38%) to good (45%) to moderate (17%) for RT (Fig. 6 H). In 17% of cases, the scores were equal, whereas the difference in combined clinical score was less than 0.5 in 48% and less than 1 in 82% of cases. In only one patient, edge definition and motion artifact were scored as poor for the RT sequence. The difference between clinical scores for the BH and RT sequences was statistically significant (*p* < 0.0001). Figure 7 shows representative BH and RT images from patients with a good to excellent clinical score for BH.

## Discussion

The results from the two-center clinical study show that RT-bSSFP yielded diagnostic image quality cine images in all 60 patients encompassing a wide range of heart rates (43–116 bpm), respiratory rates (12–20 bpm), and ventricular indices (LV end-diastolic volume 84–397 mL, LV end-systolic volume 35–325 mL, LVEF 18–71%) with spatial, temporal, and contrast resolutions that were comparable to BH-bSSFP. To the best of our knowledge, this is the first study that has performed clinical validation of the free breathing cine bSSFP imaging in a large cohort of adult patients. The bias and limits of agreement between RT and BH sequences for LV and RV volumetric indices and LV mass were comparable to inter-observer variability in both RT and BH sequences. An interesting observation was that while the LV volumes were slightly overestimated (mean bias of  2–3 mL) with BH sequence compared to RT sequence, RV volumes were slightly underestimated (mean bias of 2–3 mL). Although such small differences in ventricular volumes may not be clinically significant, it may be worth investigating if the intra-thoracic pressure differences between respiratory suspended state and free breathing could be a contributing factor.

There are several key findings from the study that are worth noting. Firstly, conventional respiratory triggering methods for circumventing the breath-holding in bSSFP imaging that do not account for signal intensity modulation during the approach to SS can introduce significant artifacts as demonstrated in this study. Interestingly, even in BH bSSFP acquisitions, through-plane motion of the base toward the apex of the ventricle during systole introduces quantifiable temporal signal heterogeneity in the myocardium. Compared to other previously published respiratory triggered bSSFP cine CMR implementations [21, 23], RT sequence implementation [20, 22] mandates attainment of M_ss_ prior to cardiac gated data acquisition. Metrics to quantify the temporal stability of the signal demonstrated that the RT sequence attained SS in synchrony with the physiologic cycle that is comparable to BH bSSFP. Importantly, the proposed acquisition technique and image quality evaluation metric have the potential to be used more broadly in other clinical applications. Also, it is worth noting that RT-bSSFP cine sequence is inherently compatible with prospective arrhythmia rejection and retrospective cardiac gating that are typically used with BH techniques, allowing the acquisition of a complete cardiac cycle. Furthermore, the cardiorespiratory synchronization approach can be seamlessly combined with other techniques such as non-cartesian sampling, sparse sampling, and self-navigation.

Secondly, bSSFP cine acquisition is feasible with both respiratory triggering using bellows and respiratory triggering with slice tracking using diaphragmatic RF navigators [20]. One advantage of using diaphragmatic RF navigators is the ability to track slice location in real time from the navigator information. However, the relatively long duration (at least one RR interval) between the slice position measurement from RF navigators and the completion of cine data acquisition may diminish this potential benefit. Furthermore, the quality of RF navigators may be compromised in patients with significant iron overload [23]. In contrast to RF navigators, respiratory bellows do not provide a direct measure of the lung-diaphragmatic interface. However, respiratory bellows provide the ability to monitor respiratory motion continuously and independent of CMR data acquisition without incurring additional RF dose.

Thirdly, respiratory triggered cine bSSFP sequences have several strengths. In contrast to continuous multi-NSA sequences which are often used to obtain cine bSSFP images in sedated patients or those with compromised respiratory function [22], the RF duty cycle in these sequences remains at about 50%, mitigating the specific energy deposition concerns and permits data acquisition over a longer scan duration. Freedom from the breath-hold constraint further allows one to attain bSSFP cine images with higher spatial resolution, temporal resolution, or coverage. Therefore, this technique has the potential to broaden the scope of clinical applications for cine bSSFP sequences, such as assessing diastolic function by obtaining a high temporal resolution dataset.

Another advantage of the RT bSSFP cine sequence is that it would reduce the potential for errors in estimation of ventricular chamber volumes due to inconsistent breath-holds. Although CMR is considered the gold standard for the estimation of ventricular chamber volumes, inconsistencies in the level of the diaphragm across breath-holds for each slice can result in under and/or over sampling of the myocardium in the direction of the ventricular long axis. This too may lead to variability in calculated volumes and EF, especially sampling-dependent inclusion or exclusion of the basal slice in end-systole [30]. This problem would be significantly diminished with free-breathing acquisitions [19].

Although we made no attempt to include only patients with compromised respiratory function, our clinical results suggest that the reduction in image quality of RT bSSFP was only small compared to the image quality attainable with the BH bSSFP technique. Notably, in our previous study, the image quality obtained with the RT-SSFP technique was better than that obtained with the multiple signal averaging that is routinely used in sedated pediatric patients [22]. In this two-center study in adults, our findings with respect to LV mass and ventricular volumetric results obtained with the RT and BH sequences are in line with the inter-observer variability.

The current implementation of RT-SSFP cine sequence has two key limitations. First, image quality of RT-bSSFP, while good, was inferior to that of the BH-bSSFP sequence. In 10/60 subjects, RT-SSFP image quality scores were moderate (still diagnostic) and we speculate that this reduction in image quality may be partly because of inconsistent respiratory patterns, such as when a patient starts inspiration before data acquisition from the expiratory trigger has completed, or respiratory drift over the course of an examination. The extent of the image quality degradation is dependent on the location of the acquisition segment with respect to center of the k-space. The image quality of the RT sequence could be improved by imposing an additional prospective constraint for rejecting data that were acquired during such respiratory inconsistencies at the cost of increasing scan time. A second limitation of the RT-bSSFP sequence is that it takes on an average 55% more time than the conventional cine BH-bSSFP sequence. For a cardiac cine bSSFP technique that requires n shots for a complete data acquisition, the RT-bSSFP sequence takes the time required for n respiratory cycles, whereas the BH-bSSFP sequence takes the time required for n + 1 heartbeats (1 extra beat to drive to M_SS_). This is because the RT sequence, in its current implementation, acquires data for a single cardiac cycle after each respiratory trigger. Often, the expiratory phase of the respiratory cycle is longer than one cardiac cycle and may permit data acquisition for several cardiac cycles, which would reduce the acquisition time of the RT sequence. Although this was not considered in this study, data acquisition over consecutive cardiac cycles during expiration with prospective constraint for rejecting the data are an important area of future investigation [31] as respiratory bellows permits continuous and simultaneous tracking of the respiratory motion.

## Conclusion

In conclusion, we have shown that the RT sequence is a robust, free-breathing alternative to BH-bSSFP for evaluating ventricular function in patients with impaired breath-holding capacity and/or arrhythmia. The RT sequence yields cine images with contrast and spatio-temporal resolutions that are identical to those attained by using the BH sequence, albeit with a modest time penalty and a small reduction in image quality. We believe that the elimination of the BH constraint on a cine bSSFP sequence has the potential to improve the evaluation of ventricular function in subjects with compromised respiratory capacity with small reduction in image quality. Moreover, this would allow one to acquire high temporal resolution cine images necessary for the assessment of transient phenomena, such as isovolumic relaxation time, as well as the ability to acquire multi-phase, high-resolution anatomic images, such as coronary MR angiography.

## Abbreviations

M: magnetization
SS: Steady state
BA: Bland-Altman analysis
BH: Breath-hold
BMC: Blood-myocardial contrast
bSSFP: Balanced steady-state free precession
CMR: Cardiovascular magnetic resonance
EDV: End-diastolic volume
EF: Ejection fraction
ESV: End-systolic volume
LV: Left ventricle/left ventricular
NI: Normalized signal intensity
PCC: Percent duration of the cardiac cycle
RF: Radio frequency
RG: Respiratory gated
ROI: Region of interest
RT: Respiratory triggered
RV: Right ventricle/right ventricular
SENSE: Snesitivity encoding
SI: Signal intensity
SIV: Signal intensity variation
SNR: Signal-to-noise ratio
ss: Steady state
TE: Echo time
TR: Time
VCG: Vector electrocardiogram
